# Appraisal of a Hypothesis on Embryo Fusion and Individuation

**DOI:** 10.3390/biology15100767

**Published:** 2026-05-11

**Authors:** Francis J. O’Keeffe, George L. Mendz

**Affiliations:** School of Medicine, The University of Notre Dame Australia, Sydney, NSW 2010, Australia; francis.okeeffe@nd.edu.au

**Keywords:** embryo fusion, human embryo experimentation, tetragametic individuals, individuation, sesquizygosis

## Abstract

Aim: Human beings comprising two genetically different types of cells are known as tetragametic individuals. This trait is thought to arise from the fusion of two embryos. This investigation into the hypothesised fusion potential of two embryos was conducted to appraise whether embryo fusion is a correct explanation for tetragametic chimerism and an appropriate basis for legislation governing human embryo experimentation. Materials: The reported hypotheses to explain embryo fusion. Fifty documented instances of human beings comprising two genetically different types of cells scattered throughout the body. Data from studies of sesquizygotic twins with key information describing embryos with two genetically different types of cells. Methods: The development of the embryo fusion hypotheses was examined. The correctness of three hypotheses of embryo fusion was appraised. Multiple reports of tetragametic human beings were reviewed. In each case, the maternal component of the genetically different cells was analysed to ascertain the presence of identical or dissimilar contributions to the embryo genome and thus establish whether one or more oocytes were fertilised. An investigation of sesquizygotic twinning was employed to assess the possibility of dispermic fertilisation generating tetragametic individuals. Results and Conclusions: The embryo fusion hypotheses have not been demonstrated, and their analyses suggest that they propose highly unlikely/impossible biological events. Individuals with identical maternal contributions incorrectly were assumed to originate from the fusion of embryos. Sesquizygosis is a plausible mechanism by which a human embryo comprised of two genetically different types of cells could be generated.

## Simple Summary

Human fertilisation generates a single-cell zygote with a unique human genome. The rare observation of human beings with two genetically different types of cells led to the hypothesis that at an early stage of development, two human embryos would have fused forming a single embryo with two genetically different types of cells. This fusion potential was postulated to exist up to the fourteenth day of embryogenesis. It was proposed that whilst a human embryo has this potential, it is not an individual. Thus, owing to this lack of individuality, it has been proposed that the legal protections accorded to human beings do not apply to early embryos. Legislation governing the ethical treatment of human embryos is based on this understanding. The current study appraised three hypotheses that attempt to explain human embryo fusion and found evidence supporting the high improbability and in some instances the impossibility of this event. The alternative explanation to embryo fusion provided by “sesquizygosis” is discussed. Review of several cases of individuals with genetically different types of cells showed that embryo fusion was inferred and not observed, and the cases were better explained by sesquizygosis.

## 1. Introduction

The ethics of human embryo experimentation have been informed by the understanding that two early human embryos may fuse into a single viable entity and thus lack individuality [1,2,3] which is regarded as a necessary criterion for human embryos to be considered individuals and thus afforded appropriate status as human beings [4,5,6,7].

In human embryology, ‘zygote’ refers to a single cell formed through the fertilisation of a secondary oocyte by a spermatozoon and goes on to the first cleavage into two blastomeres [8]. Continued rapid mitotic division of blastomeres forms a solid ball of cells called a morula. Further development results in a blastocyst which is a hollow fluid-filled mass of blastomeres. Around day 4–5 after fertilisation, the expanding blastocyst hatches by rupturing the zona pellucida that enveloped the female oocyte and persists throughout this process and proceeds to implant itself on the wall of the uterus. This concludes the germinal stage of embryogenesis. The embryonic period begins with blastomere differentiation into three layers at gastrulation, followed by neurulation with the formation of the basic elements of the nervous system and by organogenesis with the beginning of the formation of the major organs and body structures. In the final foetal stage, the embryo develops into a foetus that grows and matures into a fully formed human being made up of cells with the same genetic makeup, excepting gametes which have only half of it [9].

On rare occasions, human beings comprise two genetically different types of cells [10,11,12]. This observation has led to the hypothesis that during human embryogenesis, two genetically different early human embryos could fuse to form a single viable entity [13,14,15]. It is argued that if two embryos can combine into a single viable one, they were not individuals; hence, during the period when embryo fusion is possible there is no foundation to treat embryos as individual human beings and offer them the protections demanded by humans until individuation is assured [16,17,18].

Advocates for human embryo experimentation have proposed that individuation is guaranteed when the combining potential of two embryos ceases at the end of the germinal stage [19,20,21]. Implantation begins at 6.5 days and concludes around day 9 post-fertilisation; in the past, this event was timed at about 14 days post-fertilisation [22,23,24]. A view used to justify legislation permitting human embryo experimentation up to day 14 of embryogenesis [25,26].

Human embryos derived from four gametes are referred to as ‘tetragametic’; it is hypothesised that they have been formed by the fusion of two previously separate embryos [27,28]. The assumption that human tetragametic embryos may be formed up to 14 days post-fertilisation can be traced back to a model presented by Hellegers [1], who later proposed the combining potential of two embryos extended to the time of implantation [29]. However, a basic problem with this model is its lack of supporting evidence to explain the formation of embryos comprising two genetically different types of cells.

The present study appraised three hypotheses that attempted to explain how two embryos could fuse into a single viable entity: (i) the polar body hypothesis, (ii) the parthenogenesis hypothesis, and (iii) the fusion hypothesis.

A case is reviewed involving dispermic fertilisation of a single oocyte which produced sesquizygotic twins [30]. Sesquizygosis occurs when a human embryo is generated by two spermatozoa that simultaneously fertilise a single oocyte, forming an embryo comprising two genetically different types of cells [31,32,33].

Analyses of several cases of individuals with genetically different types of cells showed that embryo fusion was inferred and not observed, and the cases were better explained by sesquizygosis.

**Figure 1:**
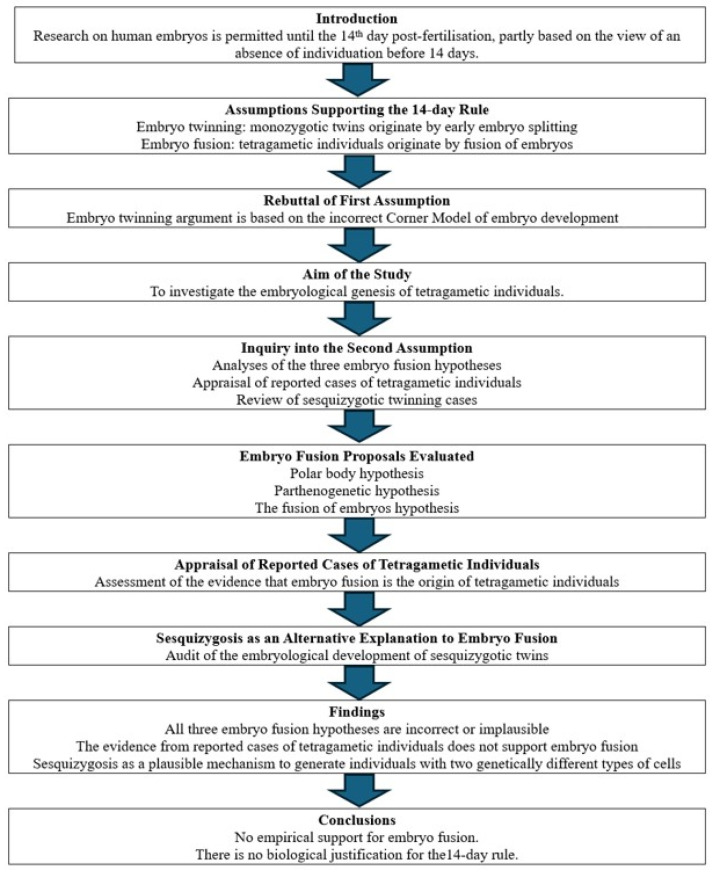
The organisation of the study.

This study suggested that embryo fusion should not necessarily be inferred from the observation of two genetically different cells within a human individual and that sesquizogosis might better explain the generation of human beings comprising two genetically different types of cells. The ethical significance of these findings is the inappropriate use of a hypothetical and unproven capacity of two human embryos to fuse together to justify in part legislation permitting human embryo experimentation up to 14 days post-fertilisation, particularly when such an event is questioned by a body of evidence. Figure 1 illustrates the structure of the study.

## 2. Observations of Genetically Different Types of Cells in Humans

The process of human fertilisation involves a sperm cell and an ovum uniting to generate a single-cell zygote that is a genetically unique product of chromosomal reassortment [34].

The process activates the zygote to undergo a series of cell divisions called mitosis, whereby a single cell divides into two daughter cells that contain the same genome [35]. Generally, the precision of mitosis gives all the cells that comprise an organism the same genome. Albeit this does not always occur, and on rare occasions, a human individual may have two genetically different cells, that is, with different genomes generated during development [36].

Ordinarily, this observation does not bring into question the individuality of a human embryo. For example, small quantities of genetically different cells have been found in human beings that derive from another individual [37]. This is observed during pregnancy when a bidirectional exchange of blood has occurred between the mother and foetus [38]. The extraneous cells that derive from fetomaternal transfers can persist for decades and could be found in various maternal tissues and organs, including the blood, bone marrow, skin and liver [39].

On occasions, the presence of genetically different types of cells in an individual is discovered in only one tissue [40]. E.g., when blood is exchanged between monochorionic dizygotic twins or the placentae of dizygotic twins [41], or following bone marrow or organ transplants [42], or after receiving a blood transfusion [43]. Nonetheless, the presence of small quantities of genetically different types of cells within a human being presents no issues for determining the individuality of their originating embryo.

Sometimes human beings have two genetically different cells broadly spread in their body tissues, and this observation has been explained by hypothesising that two individual embryos may have combined into a single viable entity [13,27,44]. The fusion potential of two early human embryos is often thought to extend up to 14 days of embryogenesis [19,20,21,24]. These cases raise the question of when the individuation of human beings is established.

Embryo fusion is not the sole basis to support legislation permitting human embryo experimentation up to 14 days post-fertilisation, but it has been used extensively to justify this rule [25,26,45]. Therefore, it is important to evaluate the evidence supporting this supposition and how it came to be accepted as a mechanism by which human beings with two genetically different types of cells are generated.

## 3. Development of the Embryo Fusion Hypothesis

The first cases reporting human beings comprising two genetically different cells were documented during the early 1960s [46,47]. These studies reviewed the generation of XX-XY human individuals and reported a double genetic paternal contribution in each patient’s cells [46,47]. The first of these investigations was unable to definitively determine the exact nature of the material contribution and concluded, “the eventual discovery of similar cases of newer techniques may provide more definitive information” [46]. However, the second study concluded, “there is no evidence of a double maternal contribution” [47].

In the mid-1960s, a hypothesis was proposed that two individual human embryos could combine into a single viable entity [48]. Also, it was suggested that a viable human embryo could be generated by fusing two morulae [49]. The bases for these assertions were two animal experiments performed during the early 1960s, which reported a fusion of two 8-cell mouse embryos into a single viable entity [50,51]. The authors provided no rationale for applying these data to human embryos.

The success of these experiments was partly attributed to an experimental dissolution of the zona pellucida—the thin translucent membrane of the oocyte that surrounds embryos during early embryogenesis—which made the fusion possible. To note, these experiments involved embryo manipulation that cannot be performed in vivo, and there is a small probability that two embryos in close proximity will lose the zona pellucida simultaneously to make this event naturally possible. Nonetheless, after these experiments involving nonhuman mammals, embryo fusion was assumed to be a mechanism to generate individuals with two genetically different cells [52].

In 1969, a literature review was conducted on reports of human beings with two genetically different cells; it concluded that no evidence had been discovered that conclusively established that two human embryos could fuse into a single viable entity [53]. This caution was followed by an article on spontaneous chimerism in mammals, which stated:

“Since critical studies of early zygotic errors are not yet available in man, and because none of the cases so far described allows a definitive conclusion, it is premature to discuss the likelihood of one type of normal event or the other as leading to whole-body chimerism in man”.

[54]

Despite this caution, in 1970 a very influential article was published maintaining the idea that two separate human embryos may spontaneously fuse together to form a single viable entity: “Less well known is the fact that it is also in these first few days [of embryogenesis] that twins or triplets may be recombined into one single individual” [1] (Italics added). The publication did not specify up to what point in time embryo fusion was possible [1], and yet in 1973 a timeframe was added:

“During the first week following fertilization, it’s not clear whether one or more lives will result, so that not until implantation can one begin to talk about irreversibly individual human life, that is to say, the point beyond which one can no longer be combined with another individual to become one child”.

[29]

The time of implantation was correctly considered at the end of week 1 post-fertilisation [55], and three reasons were proposed to support embryo fusion to be possible up to this time [1]: First, by citing experiments performed in the early 1960s that involved two mouse embryos which were experimentally combined into a single viable embryo [50,51], and extrapolating the data involving mouse embryos to human embryos. Secondly, stating from two case studies involving individuals with genetically male and female cells [54,56] that “these human so-called chimaeras, whose genetic type is XX-XY, are in fact recombinations into one human being and are the products of more than one fertilization” [1] but providing no clear reasoning for this conclusion and overlooking that both studies did determine the precise maternal contribution within the genomes of the individuals [54,56]. Lastly, by referring to a review published in 1969 that documented six cases of human beings with two genetically different cells [1]. Notably, three of these studies concluded that fertilisation of a polar body, which subsequently fused with a human embryo best explained the observation of human beings comprising genetically different cells [52,57], without clarifying the nature of the maternal genetic contribution, that is with insufficient evidence.

Two cases in this review proposed that two separate human embryos combined to form a tetragametic embryo [52,57]. However, embryo fusion was not demonstrated; it was merely asserted based on the observation of two genetically different cells within human individuals. The final case mentioned in the article offered no explanation for the generation of humans comprising two genetically different cells [58,59].

Hellegers never demonstrated that two human embryos may fuse together. Therefore, it must be investigated whether any evidence, beyond the studies Hellegers cited, proves that embryo fusion may occur. Since 1962, several cases have documented human beings with two genetically different types of cells. Over time, three hypotheses emerged to explain how two human embryos could fuse into one viable entity.

## 4. Appraisal of Three Hypotheses of Embryo Fusion

In embryology, the generation of a female gamete proceeds through meiosis, two cell divisions that result in a haploid oocyte or ovum. During each of the two meiotic divisions, a much smaller cell called a polar body forms with its much larger sister oocyte and is extruded from it [60]. Polar bodies generally undergo cell death by apoptosis 17–24 h following their formation [61].

The first meiotic cell division produces a secondary oocyte and the first polar body (PB1) [62]. Generally, PB1 do not undergo any further cellular division [63,64], although on occasions it may divide into two daughter polar bodies [65]. The secondary oocyte continues its development until it arrests at a meiosis stage called metaphase II [66]. Meiotic cell division only recommences if a spermatozoon penetrates this oocyte, a process that results in a mature ovum and a second polar body (PB2) that is also expelled from the ovum [67].

The existence from 1962 onwards of 50 cases of human beings with two genetically different types of cells was documented in 2020 [28]. In many of the studies reviewed, researchers concluded that genetic similarities in the maternal contribution to the genetically distinct cells ruled out the possibility that two genetically distinct female gametes were fertilised prior to the occurrence of embryo fusion [47,68,69]. In response, two hypotheses were proposed to explain how embryo fusion could produce a single embryo comprising two genetically distinct cells that indicated the involvement of a single maternal contribution.

The first hypothesis suggested penetration of a spermatozoon into a polar body, generating a zygote which then merged with a human embryo that resulted from fertilisation of a secondary oocyte constituting a single embryo comprising two genetically different types of cells [70,71,72]. Here, ‘fertilisation’ refers to the series of events that end with the formation of the zygote.

The second hypothesis proposed that human parthenogenesis could activate a single female gamete to produce two identical maternal haploid nuclei. These two haploid nuclei are each individually fertilised by different spermatozoa to produce two zygotes, which merge to form an embryo with two genetically distinct cells [73,74,75].

In a small number of the 50 human beings reported, it remained uncertain whether identical or different maternal genetic contributions are found in the genomes comprising two genetically different types of cells [28]. To address the possibility of different maternal contributions, a third hypothesis proposed that two individual human embryos may fuse together [76,77,78]. These three hypotheses will be examined.

It should be noted that “these models remain speculative and largely unsupported by biomolecular or cell biological data” [79].

“Taken together, these analyses point to the occurrence of human parental genome segregation errors and the persistence of these lineages to the blastocyst stage resulting into chimeric and/or mixoploid human blastocysts”.

[79]

### 4.1. The Polar Body Hypothesis

It has been proposed that human embryo fusion may happen following a hypothetical ‘polar body fertilisation’ [80]. This refers to an unobserved event whereby a spermatozoon fertilises a polar body to create a zygote [81]. On rare occasions, twins have been observed with different paternal contributions within their genomes, but the exact nature of the maternal contribution remained indeterminate [82]. To explain this observation, it has been proposed that both an ovum and a polar body were fertilised by two different spermatozoa, thus creating two zygotes [83]. These two zygotes then fuse to form a single embryo with two genetically different cells, although similarities in the maternal contribution are observed [80].

However, there is a conceptual flaw in this analysis. This hypothesis is based upon an incorrect assumption that a polar body and the ovum from which it was extruded are genetically similar to each other. In fact, female meiosis produces daughter cells (an oocyte and a polar body) that contain many genetic dissimilarities because of a ‘crossing over’ of genetic material that occurs during the early stages of meiosis [84]. Thus, if a polar body could be penetrated to establish a zygote capable of fusing with a human embryo, not one but two genetically different maternal contributions would be observed in the single embryo [85].

There are further difficulties with proposing that spermatozoa may penetrate polar bodies. For example, human fertilisation is initiated when a spermatozoon penetrates a secondary oocyte arrested during the metaphase II stage of meiosis [86]. The secondary oocyte is unable to complete the second meiotic division unless it is penetrated and activated by the male gamete during this stage of the cell cycle [87]. In contrast, an investigation into the physiology and behaviour of human first polar bodies (PB1) revealed that, unlike secondary oocytes, these entities do not need to be fertilised to undergo further cell division [65], and PB1 are not arrested at metaphase II.

Condensed chromatin observed in this PB1 was interpreted as it entering interphase [65]. A PB1 contains bivalent chromosomes, and if after interphase the cell cycle continued it would be a mitotic division of the polar body, and the daughter cells would have sets of chromosomes that would make fertilisation impossible. In the same study with another oocyte the corresponding extruded PB1 was undergoing division which was interpreted as meiosis II.

The study conducted by Ortiz et al. [65] investigated only the physiology and behaviour of PB1 that were not penetrated by a spermatozoon. In the event that spermatozoon penetration of PB1 is observed, a series of events may indicate whether successful fertilisation is achievable. If the DNA replication is not accompanied by a replication of the non-genetic cytosolic content, the daughter cells will be minuscule (relative to oocytes) and it is highly unlikely that they would have the cellular machinery necessary to achieve the DNA manipulation events that generally follow spermatozoon penetration.

A difficulty with proposing the successful fertilisation of a polar body is that polar bodies contain reduced quantities of cytoplasm, which may inhibit a series of events necessary for fertilisation to occur [88]. It could be argued that polar bodies contain a sufficient concentration of cytoplasm necessary for fertilisation. This cytoplasm would need to contain a large quantity of enzymes, required to arrange the sperm and ovum pronuclei to make fertilisation possible [89]. Further investigations are required to ascertain whether polar bodies are capable of embryonic development.

Moreover, during fertilisation, after a spermatozoon has penetrated the extracellular coat of the ovum, it interacts with its plasma membrane overlying the tips of the microvilli of the ovum surface. Neighbouring microvilli then rapidly elongate and cluster around the sperm to ensure that it is held firmly so that it can fuse with the ovum. However, updated investigations into fertilisation reveal that the region of the ovum where the first polar body is located does not contain microvilli [34]. Such a finding may indicate that successful penetration of PB1 by spermatozoon is a rare occurrence.

Furthermore, investigations into the physiology and behaviour of PB2 have only documented polar bodies undergoing cell death (apoptosis) when contacted by sperm [90]. Thus far, the creation of a viable human zygote generated by the merging of sperm and polar body 1 or 2 has never been observed [91]. This has led some researchers to propose that it does not happen [85]. Currently, there is a need to demonstrate that a zygote can be generated when a spermatozoon fertilises a polar body. Until this is established it should not be proposed that embryo fusion occurs via the fusion of a human embryo and a spermatozoon fused to a polar body.

### 4.2. The Parthenogenetic Hypothesis

Parthenogenesis refers to an asexual reproduction in which without spermatozoa to initiate fertilisation, a spontaneous activation of a secondary oocyte results in a female producing a viable embryo capable of full development into normal offspring. It is observed in some animal species [92], but human parthenogenesis has never been documented other than in a few cases when parthenotes have been considered embryos [93]. A mechanism for presumed parthenogenesis in humans proposes that “the secondary oocyte and first polar body enclosed in the zona pellucida may fuse together to form a single cell that restores the diploid number of chromosomes and initiates cell division” [93]. Other than suggesting a mechanism, there is no support for this explanation. On rare occasions, cellular growths such as teratomas have been observed in human patients which are attributed to an ‘activation’ of a secondary oocyte [93]. Nonetheless, these reports do not demonstrate that a viable human embryo can be generated without spermatozoa.

A hypothesis to explain the generation of human beings with two genetically different cells suggests that human parthenogenesis could activate a single female gamete to produce two identical maternal haploid nuclei [94]. In the event that these haploid nuclei are simultaneously fertilised by different spermatozoa, an embryo is formed with two genetically distinct cells that contain an identical maternal genetic contribution [95].

This hypothesis confuses parthenogenesis with endoreduplication. In human biology, endoreduplication is a cell cycle variant where a cell replicates its genome without undergoing complete mitosis, leading to polyploid cells [96]. If a female gamete could replicate its genome to form two haploid nuclei, such an occurrence would indicate endoreduplication and not parthenogenesis.

Endoreduplication is a mechanism of the developmental programme in which mitotic cell cycles switch to endocycles resulting in polyploidy, that is, cells with more than one genome. This mechanism has been observed in vitro in mouse trophoblast stem cells triggered by depletion of fibroblast growth factor 4, differentiating into polyploid trophoblast giant (TG) cells essentially involved in implantation and placentation. The production of multinucleated TG cells could explain the pre-eclampsia and placentomegaly observed in mice and humans [97].

Endoreduplication has never been observed in the human germline. Nonetheless, assuming the possibility of gamete endoreduplication, a polyploid gamete fertilised by a single spermatozoon would yield blastomeres with identical genomes leading to individuals with identical genome cells, not tetragametic individuals. If two spermatozoa could simultaneously penetrate a single ovum which contains two genetically identical nuclei generated by endoreduplication, the formation of two different zygotes with (i) aligned polarisations and (ii) requiring the segregation of the interaction of each paternal pronucleus with each one of the maternal pronuclei, creating two separated astral microtubules enveloping each pair of paternal and maternal pronuclei. These are highly unlikely events within the confines of the zona pellucida. At variance with the polar body hypothesis, fusion of these hypothetical zygotes would result in a tetraploid individual with identical, not just similar, maternal contributions to the genomes of the two genetically different types of cells, a case that has never been reported.

Thus, the parthenogenic hypothesis is based on speculative unlikely events for which there is no evidence: the endoreduplication of gametes, the formation of two zygotes within the zona pellucida and the fusion of those zygotes into a viable embryo.

### 4.3. The Fusion Hypothesis

It has been proposed that many instances of human beings with two genetically different types of cells are generated by the fusion of two separate embryos with different maternal and paternal genetic contributions into a single viable organism [13,14]. Several possible events have been suggested: (1) two zygotes may combine to form a single entity [15]; (2) two 2-cell human embryos may fuse together to form a single organism [98]; (3) two morulae may amalgamate into one embryo [71]; and (4) two blastocysts could merge to establish a single embryo [99,100], (a) up to implantation [101], or (b) during the first 14 days of embryogenesis [7,24], or (c) in the third week of pregnancy [102]. These proposals are hypotheses to explain observations; they are not observations themselves, and there are several problems with them. A summary of the difficulties with this hypothesis is given in Table 1.

The suggestion that two human zygotes with different maternal and paternal genetic contributions may combine to form a tetragametic embryo [15] must be discarded because the fusion of two zygotes would produce a polyploid cell comprising a large excess of chromosomes. In most instances, human polyploidy cells arrest development and are unviable resulting in spontaneous abortion, and in the extremely rare case of liveborn infants their survival is only a few days [103,104].

When a human spermatozoon penetrates the zona pellucida, it triggers a series of chemical reactions that harden this membrane preventing multiple sperm from fertilising the ovum [105]. The zona pellucida functions to prevent polyspermy and embryo fusion in the event that two ova are simultaneously fertilised [106,107]. A hardened zona pellucida surrounding each of the two embryos would prevent embryo fusion.

A different situation is the dispermic fertilisation of conjoined oocytes from binovular follicles or joined follicles that share a common or blended zona pellucida. It has been hypothesised that each zygote may develop individually into a morula whose blastomeres eventually mingle to form a single embryo. Studies of early cleavage in the fertilisation of single human female gametes show that errors such as chromosome segregation and multinucleation occur with high frequency during the first mitosis and result in a lower blastocyst developmental rate [108]. Thus, a fundamental problem with the explanation of the dispermic fertilisation of two oocytes that share a common zona pellucida is that it assumes that the four pronuclei from the two spermatozoa and the two ova will interact in segregated male-female pairs yielding separate zygotes that independently would cleave into pairs of blastomeres. A central objection to this interpretation is that to prevent genetic defects, an accurate first mitosis is essential and it occurs predominantly orthogonal to the axis of juxtaposed pronuclei. The process of orienting the mitotic spindle relative to the plane of cell division ensures accurate segregation of chromosomes and is linked to correct development to the blastocyst stage [109]. In the dispermic fertilisation discussed the direction of each zygotic cleavage is spatially controlled and would demand an alignment of the two pairs of pronuclei almost impossible to occur.

Thus, it remains to appraise the reported cases of tetragametic individuals with different maternal contributions proposed to originate through the merging of morulas that result from the development of two human zygotes each enveloped in its zona pellucida. The process will require a melding of these membranes that under natural conditions have not been observed in humans. This basic problem will also apply to the proposed explanations of cases of dispermic fertilisation of two ova, or an ovum and a polar body followed by their amalgamation.

Multicellular embryo amalgamation needs more than the dissolution of the zona pellucida. Following morula compaction, blastomeres are differentiated into two cell lineages: the outer polar cells that form the trophectoderm outer layer of the embryo and the inner apolar cells forming the embryoblast [110]. To create a viable entity, the merging of both embryos must be such that each type of differentiated blastomere mixes with its equivalent type in the other embryo, an event with extremely low probability. For example, embryo fusion requires reciprocal adhesivity of trophoblasts between the two embryos, a property that diminishes markedly in the morula-blastocyst transition, even before the beginning of the blastocoele formation [111]. This decreased adhesivity is attributed to tight junctions that form between trophectoderm cells and impede the fusion of two individual morulae in human embryo experiments [112].

The natural spontaneous fusion of two human blastocysts is a rare event, although it has been observed with greater frequency in embryo group cultures [113]. During embryogenesis, the human blastocyst hatches from the hard zona pellucida around days 4–5 [114]. Two hatching blastocysts whose trophodectoderm layers meet could merge into a single structure with two inner cell masses. In a study with over 3000 in vitro cultured blastocysts, complete trophectodermal amalgamation between two hatching blastocysts with the formation of dizygotic twin blastocysts was observed on two separate occasions and no blastocyst was observed that would lead to a single tetragametic individual [115]. These results support the view that natural blastocyst fusion in the female genital tract would lead to extremely rare monochorionic dizygotic twins rather than extragenetic individuals. In addition, fusion of two blastocysts in the female genital tract necessitates an improbable collision between two embryos prior to implantation, an unlikely event considering that in half of the cases the embryos would enter the uterus through separate Fallopian tubes [116]. The two blastocysts would then have to collide with incredible precision at the same point on the endometrial surface. The enormous lack of proportion between the surface area of the lining of the uterus and the size of each embryo makes such a collision statistically improbable.

Lastly, the fusion of two implanted human embryos in week 2 of embryogenesis is unlikely because it would involve coordinated action of molecular factors, modified endometrial structures, and two bilaminar or trilaminar embryos with increasingly complex structures to act in the right sequence and to combine with an impossible precision to merge their individual cell populations [116,117]. Human embryo implantation is generally timed beginning about 6.5 days and concluding on day 9 post-fertilisation [55]. Presently, no hypothesis can explain the fusion of two fully implanted human blastocysts. After day 9 of embryogenesis, the human embryo is fully implanted in the thick endometrium, which would likely impede the fusion of two human embryos.

Other considerations suggest that human beings with two types of genetically different cells may not be generated by embryo fusion. Triploidy is one of the most frequent chromosomal errors responsible for reproduction failures. An analysis of several findings argues that many tetragametic individuals result from triploid dispermic zygotes that undergo postzygotic diploidisation which is the origin of unusual cases of mosaicism and twinning [118].

## 5. Sesquizygosis and Human Chimeric Individuals

Sesquizygosis is a rare form of fertilisation that occurs when two spermatozoa simultaneously fertilise a single oocyte to generate a human embryo with two genetically different cells [30,33]. It can be verified by genetic testing and is indicated by a single maternal and double paternal contribution in the genetically different cells that comprise a single human being [31,33]. Sesquizygosis produced an XX-XY human embryo which divided and produced twins of different sexes. This event may help to explain the generation of human beings comprising two genetically different types of cells.

In 2014, a woman initially thought to be pregnant with identical twins was found to carry two foetuses of different sexes. Genetic testing showed that each twin comprised two genetically different cells: one type with two X chromosomes and the other with X and Y chromosomes. It was further confirmed that a single maternal and double paternal contribution comprised the twins’ genomes [30].

Two explanations were examined. First, whether a fertilised polar body fused with a human embryo to generate a human embryo with an XX-XY genotype, which then divided to establish twins who each contained genetically male and female cells [30]. This hypothesis was dismissed because a polar body is genetically different from the oocyte from which it is expelled [85], and therefore, two maternal contributions would be observed in the twins’ genomes, not one.

Second, it was considered whether human parthenogenesis could generate these twins. A dispermic fertilisation of an oocyte that underwent endoreduplication will not involve an embryo fusion that results in chimeric individuals but only one individual [30].

It was concluded that dispermic fertilisation of a single ovum best explained the presence of XX and XY cells within the sesquizygotic twins [30]. This proposal was made in light of discoveries in mammalian embryology suggesting that on rare occasions, a dispermic fertilisation of a single oocyte produced two genetically different types of cells, which were generated according to a process called ‘heterogoneic cell division’ [119].

The observation that ‘dispermic fertilisations commonly segregate paternal genomes into different cell lineages’ [30] was interpreted as proposing that the chromosomal contents of the single ovum and the two sperm formed a tripolar spindle apparatus prior to the first cleavage division, which produced two genetically different cells that comprised a single embryo [30]. This embryo then subsequently divided to establish twins of different sexes.

## 6. Review of Cases of Tetragametic Human Individuals

Fifty cases of human beings with two genetically different types of cells were documented between 1962 and 2020, and it was assumed that embryo fusion best explained their genotype. The article by Madan [28] references these investigations with their databases.

If two human embryos could merge to form a tetragametic embryo, then genetic analyses would identify two different maternal and paternal contributions in the genomes of individuals. Thus in tetragametic individual case studies discussing embryo fusion, it must be considered whether they conclusively established the presence of two maternal and paternal genetic contributions.

Tetragametic individuals are sometimes described as ‘’chimaeras’ without reference to the possible origin of the two or more types of cell populations with different genomes. Yet, consideration of the origin is important because mosaicism arises from a single fertilised ovum which through chromosomal or genetic mutations develops genetically different types of cell populations. On the other hand, chimerism proper is proposed to result from the fusion of two or more distinct zygotes or embryos. Recognition of the different usage of the term ‘chimera’ is necessary to appraise the reality of embryo fusion.

The origin of mosaic tetragametic human beings with identical maternal contributions can be explained by the fertilisation of two spermatozoa of a single ovum generating a zygote with genetically different blastomeres. The mechanism would be similar to that proposed for sesquizygosis but leading to a single individual not two.

The origin of chimeric tetragametic individuals with different genetic maternal and paternal contributions is proposed to result from the fusion of two or more distinct zygotes or embryos leading to tetraploid entities never observed to be viable in humans.

In the Vanishing Twin Syndrome one or more foetuses in a multiple gestation disappear during early pregnancy and the tissues are reabsorbed by the mother or the surviving twin(s); the mother may experience minimal or non-specific symptoms. Before advances in ultrasonography the Syndrome went undiagnosed in early prenatal imaging. Epidemiological studies estimate that the Syndrome occurs naturally in 15–36% of twin pregnancies and 30–50% in pregnancies with more than three foetuses and in 20–30% of pregnancies achieved through assisted reproductive techniques [120]. Thus, in cases of tetragametic individuals, the occurrence of this event during pregnancy must be ruled out to conclude that chimerism proper resulted from embryo fusion. In the cases reviewed the absence of a vanishing twin was not established.

Published cases reporting on tetragametic human beings refer to studies conducted on individuals with XX-XY genotypes where evidence was found for sex chromosome disorders and/or disorders of sex development that were attributed to embryo fusion [76,97,121,122,123,124,125,126,127]. Moreover, in some instances only partial maternal genetic similarity was reported [47,68,69]. These cases demonstrated the existence of tetragametic persons but provided no evidence to support that it originated from embryo fusion. Table 2 provides a summary of the problems found in 11 case studies.

## 7. Discussion

The hypothesis of the fusion of embryos originated in the mid-1960s, stating that two individual human embryos may merge into a single viable entity [47,48]. The spontaneous fusion of two human embryos to constitute a single ‘tetragametic’ entity has not been demonstrated; it has only been inferred based upon the observation of two genetically different types of cells within human beings. Also, the hypothesis was partly based upon experiments that combined two mouse embryos, achieved via procedures that cannot be replicated in vivo and are inapplicable to human beings [49,50].

The mechanism of embryo fusion was promoted by assuming a capacity of two individual human embryos to merge into a single entity up to the point of implantation [1,29]. The observation to support this assumption was the existence of human beings with an XX-XY genotype and the conclusion that this genotype could be generated only if embryos fused together [1].

Three mechanisms of embryo fusion that lack empirical support have been advanced to explain the existence of tetragametic individuals [28]. Detailed appraisal of these models employing current embryological knowledge led to the conclusion that they are highly improbable or impossible.

Sesquizygosis has significant implications for understanding the generation of human beings comprising two genetically different types of cells [128]. It demonstrated that two spermatozoa can simultaneously penetrate an ovum and generate a single embryo with genetically male and female cells that developed into viable twins of different sexes. It illustrates that the chromosomal contents from two sperm cells and a single ovum can rearrange themselves to form two genetically different types of cells that comprise a single embryo providing a plausible explanation for the single maternal genetic contribution often identified in the genomes of human individuals comprising two genetically different cells. The existence of sesquizygotic twins required a re-evaluation of the inference of embryo fusion from observations of XX-XY genotypes and offered an alternative explanation.

By 2020, there were over fifty cases of human beings with two genetically different cells scattered throughout their bodies [28]. Many of these studies verified the presence of a similar maternal genetic contribution in the genotype of these human beings; the present work argued that these cases cannot be explained by proposing the fusion of fertilised human oocytes or polar bodies, or zygotes or embryos because such events could not produce tetragametic individuals with different types of cells that contain only a single maternal contribution. Alternatively, dispermic fertilisation of a single ovum may explain why human beings with two genetically different types of cells have a single maternal contribution in their genome.

Review of the cases involving tetragametic individuals with different maternal and paternal contributions showed that the conclusion that they originated from embryo fusion was not based on empirical observations but simply inferred.

The maternal contribution to the genetically different types of cells in tetragametic individuals is central to determining whether the origin of their genome was one or more ova or polar bodies. If the maternal contribution were identical, the origin would be a single ovum. In future studies, the maternal contribution must be established unambiguously.

The existence of tetragametic individuals does not necessarily demonstrate embryo fusion. Embryonic analyses are essential to determine the presence of more than one embryo, which is the proper way to gather evidence supporting fusion, and not its simple postulation from observations in human beings. Thus, future studies need to establish the pre-existence of two embryos.

Further investigations into sesquizygosis will serve to ascertain if this twinning mechanism better explains tetragametic individuals.

## 8. Conclusions

This work questions the hypothesis of embryo fusion, which has been accepted as factual since the 1960s. In addition, the practical significance of this study is the proposal that sesquizygosis might be an alternative mechanism to explain the observation of human beings comprising two genetically different types of cells.

The spontaneous fusion of two fertilised human oocytes or polar bodies, or embryos into a single viable entity is yet to be demonstrated. Analyses of the stages of embryo development from the zygote to embryo implantation before which this event might take place indicated multiple problems and barriers that make this mechanism of generating tetragametic individuals highly improbable or impossible.

Presently, there is no firm biological reason to accept that two human embryos can fuse into a single viable organism. The ethical significance of these findings is that legislation permitting human embryos up to 14 days post-fertilisation should not be based upon an unproven fusion capacity of human embryos that undermines their individuality.

## Figures and Tables

**Table 1:** Difficulties with the embryo fusion hypothesis.

Stage of Embryogenesis	Problems with Embryo Fusion
Fusion of Two Zygotes	Produces a polyploid cell with excess chromosomal content. The zona pellucidae surrounding both zygotes would likely prevent embryo fusion before hatching.
Dispermic Fertilisation of Binovular Follicles in a Single Zona Pellucida	Four pronuclei are unlikely to interact by pairs in segregated regions yielding zygotes that independently would cleave into pairs of blastomeres with different genomes.
Fusion of Two Morulae	The embryoblasts, trophoblasts and blastomeres comprising two different embryos would have to amalgamate with their own kind of each embryo. Embryo fusion requires reciprocal adhesivity between trophoblasts, a property that diminishes markedly in the morula-blastocyst transition.
Fusion of Two Blastocysts	Natural blastocyst fusion would likely produce monochorionic dizygotic twins, not a tetragametic embryo. It is unlikely that two blastocysts from different Fallopian tubes could collide with sufficient precision to cause an embryo fusion event.
Fusion of Two Implanted Blastocysts	Implantation begins at 6.5 days post-fertilisation and concludes about day 9 of human embryogenesis. No hypothesis explains the fusion of two fully implanted human blastocysts. During the second week of embryogenesis, the highly complex structure of the blastocyst likely impedes the possibility of embryo fusion.

**Table 2:** Appraisal of case studies of tetragametic chimerism.

Case Study	Lack of Evidence for Tetragametic Chimerism
Giblett et al. 1963 [47]	No evidence of double maternal genetic contribution.
Fitzgerald et al. 1979 [68]	No evidence of double maternal genetic contribution.
Minowada et al. 1982 [121]	The maternal double genetic contribution was alleged only after observing Q-fluorescent markers on chromosomes 13 and 22 and by alleles for the Kidd blood group system.
Schoenle et al. 1983 [69]	No evidence of double maternal genetic contribution.
Farag et al. 1987 [122]	The presence of two genetically different red blood cells alone does not establish an occurrence of embryo fusion. This is a condition called blood chimerism or hematopoietic chimerism.
Verp et al. 1992 [123]	Embryo fusion was alleged after two blood types were observed in the chimaera. This observation is better explained by proposing an occurrence of Vanishing Twin Syndrome which was not investigated.
Berger-Zaslav et al. 2009 [125]	DNA analysis from gonadal tissues was performed on 12 different chromosomes examined for an extra maternal allele. Only three loci indicated the presence of 2 paternal and maternal alleles.
Ramsay et al. 2009 [126]	Embryo fusion was inferred after more than two parental alleles were observed at some autosomal loci.
Laursen et al. 2018 [124]	DNA was extracted from washed semen. Genetic testing indicated the presence of four alleles in one locus, and three alleles were detected in three loci. However, the study also observed the presence of two alleles at most loci.
Van Bever et al. 2018 [76]	A SNP profile was conducted. Embryo fusion was inferred based only on the complex ratio of the different alleles.
de Carvalho et al., 2023 [127]	Embryo fusion was only inferred based on the XX-XY genotype.

## Data Availability

Data are contained within the article.
